# Carbapenem-resistant Enterobacterales colonisation in a tertiary PICU, Cape Town, South Africa

**DOI:** 10.4102/sajid.v40i1.720

**Published:** 2025-06-11

**Authors:** Elri du Plooy, Angela Dramowski, Pieter Nel, Noor M. Parker, Helena Rabie

**Affiliations:** 1 Department of Paediatrics and Child Health, Faculty of Medicine and Health Sciences, Stellenbosch University, Cape Town, South Africa; 2 Division of Medical Microbiology, Department of Pathology, Faculty of Medicine and Health Sciences, Stellenbosch University, Cape Town, South Africa; 3 Department of Medical Microbiology, National Health Laboratory Service, Tygerberg Hospital, Cape Town, South Africa

**Keywords:** paediatrics, paediatric intensive care, carbapenem resistance, Enterobacterales, CRE colonisation

## Abstract

**Background:**

Carbapenem-resistant Enterobacterales (CRE) are important healthcare-associated pathogens in resource-limited paediatric intensive care units (PICUs). The prevalence and clinical predictors of CRE colonisation in South African PICUs are unknown.

**Objectives:**

To determine CRE colonisation status in a South African PICU.

**Method:**

Between 01 January 2022 and 31 December 2022, we collected admission and exit rectal swabs from children admitted to Tygerberg Hospital PICU, Cape Town. Prevalent CRE was defined as CRE-colonised at PICU admission, including children isolating CRE in the preceding 6 months. Incident CRE was defined as acquisition of CRE colonisation during the PICU stay.

**Results:**

Among 638 PICU admissions, we included 552 children (median age 9 months, 54% male) with an entry swab and/or known positive CRE colonisation status; 237 (42.9%) had exit rectal swabs collected. Prevalent CRE was identified in 8% (44/552) on admission, with 29/44 (65.9%) newly identified as CRE-colonised. Incident CRE was identified in 24/227 (10.6%) admissions. Children with prevalent CRE were younger than those not CRE-colonised at PICU entry (median 4.5 months vs 10 months; *p* < 0.05). Children with incident CRE were younger (median 3 months vs 8 months; *p* < 0.05), and had longer PICU stays (median 7 vs 4 days; *p* < 0.05) compared to those who remained CRE-non-colonised.

**Conclusion:**

CRE colonisation is common in PICU patients with implications for admission, isolation and antibiotic policies. Better understanding of clinical predictors of CRE colonisation will support the development of appropriate CRE screening recommendations and interventions.

**Contribution:**

This study provides insight into the burden and predictors of CRE colonisation in a South African PICU setting.

## Introduction

The emergence and dissemination of carbapenem-resistant (CR) pathogens in healthcare settings is a major public health threat. Gram-negative healthcare-associated pathogens with substantial rates of CR include the Carbapenem-resistant Enterobacterales (CRE; notably *Klebsiella pneumoniae* and *Escherichia coli*), *Acinetobacter baumannii* (CRAB) and *Pseudomonas aeruginosa*. These difficult-to-treat pathogens are increasingly prevalent as colonising pathogens both in the community and among hospitalised patients. As a leading cause of healthcare-associated infection (HAI), CR pathogens contribute significantly to in-hospital morbidity and mortality, as well as escalating healthcare expenditure.^1,2^

Early recognition of CRE colonisation, along with the implementation of infection prevention and control (IPC) measures, is imperative to prevent inter-patient transmission and institutional outbreaks.^3^ The 2017 World Health Organization (WHO) *Guidelines for the prevention and control of carbapenem-resistant Enterobacteriaceae, Acinetobacter baumannii and Pseudomonas aeruginosa in healthcare facilities* recommend multimodal IPC strategies, surveillance of CR infections or outbreaks, surveillance cultures for colonisation with CR organisms in asymptomatic individuals and the environment, and contact precautions, patient isolation and environmental cleaning.^1^

Data on the prevalence of CRE in Africa are increasing. A recent systematic review and meta-analysis on third-generation cephalosporin-resistant and CRE colonisation among children in sub-Saharan Africa by Ruef et al. showed a pooled CRE carriage proportion of 3.6% (95% CI: 0.7% – 16.4%) in 9408 children across 40 studies.^4^

Since the report of the first South African paediatric CRE case in 2012^5^ and the first neonatal CRE case in 2015,^6^ several neonatal and paediatric studies describing risk factors for CRE colonisation and/or infection, presence of carbapenemase genes and antibiotic resistance patterns have been performed in the country.^3,7,8,9,10,11,12,13^ Between 2015 and 2018, 38% (485/1293) of CRE-positive specimens from South African tertiary hospitals were submitted from children and adolescents.^14^ Between 2019 and 2020, neonates (≤ 28 days) contributed 14.3%, infants (29 days–11 months) 10.3% and children (1–19 years) 11.0% of the positive CRE cultures from South African tertiary hospitals.^15^

In the Western Cape province of South Africa, children < 14 years contributed 34.9% (783/2242) of the total CRE isolates including 15.7% (248/1580) clinical and 80.8% (535/662) of all carriage episodes. Children (> 28 days–13 years) contributed the largest share of CRE colonisation (55.1%,365/662).^8^

Despite robust local neonatal data, there is limited information from South Africa about the prevalence and predictors of CRE in infants and older children, including the settings with potential high risk such as oncology and paediatric intensive care (PICU). We will describe the prevalence and clinical predictors of CRE colonisation in children admitted to Tygerberg Hospital (TBH) PICU in 2022.

## Research methods and design

We conducted a retrospective descriptive study on all patients with a CRE screening rectal swab admitted to TBH PICU in 2022. Entry CRE screening rectal swabs were performed as standard practice for every patient at the time of PICU admission and exit swabs on PICU discharge for children admitted for ≥ 48 h.

### Study setting

Tygerberg Hospital is a public-sector tertiary hospital in Cape Town with approximately 16 000 paediatric admissions every year. The 10-bed PICU admits ± 700 children annually. Because of space and staff constraints, most children share rooms with three other children and cohorting and isolation are usually not possible. All children are recorded in a PICU admission database.

### Study population and sampling strategy

All children admitted to PICU from 01 January to 31 December 2022 who received an entry and/or exit CRE screening rectal swab were eligible for inclusion. Children were excluded if they had no CRE screen during PICU admission and had no CRE-positive clinical isolate before or during the PICU admission.

Carbapenem-resistant Enterobacterales-colonised on admission (prevalent CRE) included children with a positive rectal swab in the preceding 6 months and newly identified CRE colonisation on admission. Acquisition of CRE colonisation during the PICU stay (incident CRE) included all children with a negative entry swab but a positive exit swab or a positive culture from a routine specimen. If a carbapenem-resistant organism was isolated on any specimen (urine, blood, sputum, tissue, fluid and cerebrospinal fluid) other than the exit or entry screening rectal swab, the positive specimen and its collection date was used as a CRE-positive exit swab, if no exit swab was performed.

Multiple hospital admissions per child were allowed, but only a single PICU admission was considered per hospitalisation episode.

Tygerberg Hospital PICU practises IPC and surveillance measures in line with WHO recommendations.^1^ This includes surveillance cultures for colonisation with CRE organisms in asymptomatic individuals and surveillance of CR infections or outbreaks, while IPC measures include contact precautions, patient isolation (as allowed by staffing and space constraints) and environmental cleaning. Infection prevention and control guidance and monitoring are performed by the Unit of Infection Prevention and Control with the assistance of a PICU-based link nurse.

Carbapenem-resistant Enterobacterales screening rectal swabs were performed by gently placing a cotton swab 2 cm – 3 cm into the rectum, rotating it up to five times, and carefully withdrawing it. The swabs were subsequently sheathed, labelled and referred to the National Health Laboratory Service (NHLS) Medical Microbiology laboratory based in Tygerberg Hospital on PICU admission and discharge (if appropriate). Samples were screened for the presence of Carbapenemase-producing Enterobacterales (CPE) by inoculating CHROMID^®^ CARBA SMART culture medium (bioMérieux, Marcy-I’Etoile, France). Results were reported as either ‘CRE isolated’ or ‘CRE not isolated’. Carbapenem-resistant Enterobacterales from diagnostic samples were identified from culture using the Vitek^®^ 2 (bioMérieux) automated identification and susceptibility testing system. Carbapenem minimum inhibitory concentrations were confirmed with the Etest^®^ (bioMerieux) method and breakpoints were interpreted according to the Clinical and Laboratory Standards Institute (CLSI) guidelines of 2020.^16^ Specific carbapenemases were not identified and strain typing was not performed.

In clinically significant CRE cases, antibiotic guidance was obtained from Infectious Diseases (ID) and Microbiology based on the antibiogram, available antibiotics and the infection site.

### Data collection

All PICU admissions during the study period were reviewed for eligibility using the PICU Microsoft^®^ Access^®^ database. Hospital and laboratory data for eligible patients were retrospectively collected and entered on a Research Electronic Data Capture (REDCap^®^) case report form. This included demographic details, CRE-related data, current admission data, including admission reason, microbiological specimen and antimicrobial agent data, as well as complications and patient outcomes. Data sources included the Microsoft^®^ Access^®^ PICU database, patient notes on Tygerberg Hospital Enterprise Content Management (ECM), and the NHLS Trakcare Lab Webview results platform.

### Data analysis

Data were analysed using Microsoft^®^ Excel^®^ for Microsoft 365, IBM^®^ SPSS^®^ Statistics version 28 and TIBCO Statistica™ version 14. The characteristics of the patients were described using standard descriptive analysis, including measures of central tendency (mean, median, proportions) and dispersion (standard deviations, inter-quartile ranges and 95% confidence intervals). Mann-Whitney U testing was used to describe measures of central tendency on non-parametric data, and Chi-square and Fisher’s Exact tests to describe categorical data.

### Ethical considerations

Ethics approval was obtained from the Health Research Ethics Committee (HREC) of Stellenbosch University (HREC reference number: N23/03/019), including a waiver of individual informed consent and permission from Tygerberg Hospital management (reference number: WC202304_028).

## Results

**FIGURE 1:**
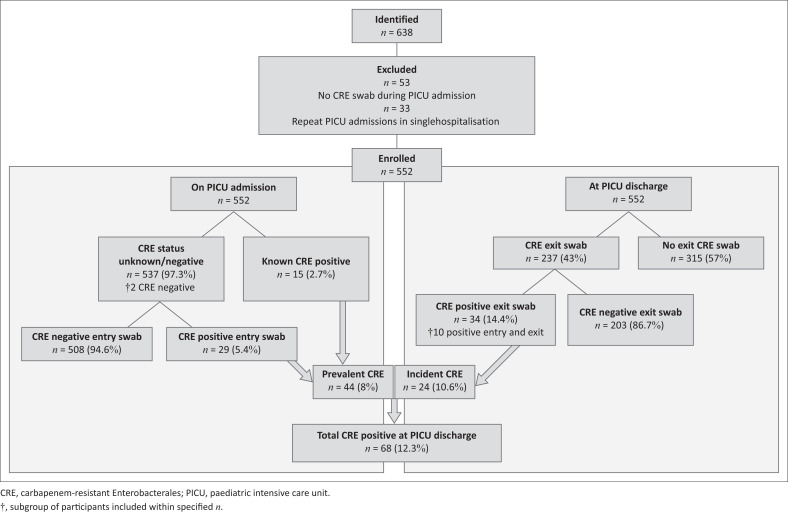
Recruitment process and carbapenem-resistant Enterobacterales status pathway.

From 01 January 2022 to 31 December 2022, there were 638 admissions to PICU, including 552 (86.5%) children who had a CRE screening swab performed on admission and/or a known CRE-positive colonisation status. A total of 237/552 (42.9%) of the admissions had an exit swab and/or a clinical specimen for CRE screening recorded (Figure 1).

Prevalent CRE at PICU entry was observed in 44/552 (8.0%), with 29/44 (65.9%) newly identified as CRE-colonised. Ten children (4.2% of 237) had a positive entry and exit swab. Incident CRE was identified in 24/227 (10.6%) patients with a negative CRE swab at PICU entry and positive PICU exit CRE swab or clinical specimen positive.

### Demographic data

Children admitted to PICU had a median age of 9 (IQR 2–39.5) months; 54% (298/552) were male. The median birth gestation was 35 (IQR 31–38) weeks, and median birthweight was 2500 g (IQR 1640 g – 3030 g). The most common reasons for PICU admission were post-elective surgical procedure (32.2%), pneumonia (24.1%) and sepsis/septic shock (11.4%). Twenty-two per cent (121/552) of children were HIV-exposed and 25 (4.5%) were living with HIV. Children were admitted to PICU for a median of 3 (IQR 1–5) days and 58 (10.5%) died during their PICU stay. See Table 1 for the profile by CRE colonisation status on PICU admission.

**TABLE 1:** Profile of children by Carbapenem-resistant Enterobacterales colonisation status on paediatric intensive care unit admission.

Variables	Total screened admissions (*N* = 552)					CRE-positive on entry‡ (*N* = 44, 8.0%)					CRE-negative on entry (*N* = 508, 92.0%)					*p*
	*N* †	*n*	%	Median	IQR	*N* †	*n*	%	Median	IQR	*N* †	*n*	%	Median	IQR	
**Age (months)**	552	-	-	9	2–39.5	44	-	-	4.5	2–9.5	508	-	-	10	3–42	0.001
< 1	552	41	7.4	-	-	44	3	6.8	-	-	508	38	7.5	-	-	0.003
1–11	552	260	47.1	-	-	44	32	72.7	-	-	508	228	44.9	-	-	-
12–59	552	146	26.4	-	-	44	4	9.1	-	-	508	142	28.0	-	-	-
≥ 60	552	105	19.0	-	-	44	5	11.4	-	-	508	100	19.7	-	-	-
Male	552	298	54.0	-	-	44	25	56.8	-	-	508	273	53.7	-	-	0.754
Birthweight (g)	249†	-	-	2500	1640–3030	34†	-	-	1910	1075–2695	215†	-	-	2550	1705–3065	0.020
Birth Gestation (weeks)	210†	-	-	35	31–38	32†	-	-	33	29–38	178†	-	-	35	31–38	0.168
Neonatal admission	395†	197	49.9	-	-	39†	29	74.4	-	-	356†	168	47.2	-	-	< 0.001
**Reasons for admission**	552	-	-	-	-	44	-	-	-	-	508	-	-	-	-	0.588
1 Post-surgical	552	178	32.2	-	-	44	21	47.7	-	-	508	157	30.9	-	-	-
2 Pneumonia	552	133	24.1	-	-	44	9	20.5	-	-	508	124	24.4	-	-	-
3 Sepsis/septic shock	552	63	11.4	-	-	44	6	13.6	-	-	508	57	11.2	-	-	-
**PICU outcome**	552	-	-	-	-	44	-	-	-	-	508	-	-	-	-	-
Duration of PICU stay (days)	551†	-	-	3	1–5	44	-	-	3	1–6	507†	-	-	3	1–5	0.540
PICU deaths	552	58	10.5	-	-	44	5	11.4	-	-	508	53	10.4	-	-	0.799
**Hospital stay**	552	-	-	-	-	44	-	-	-	-	508	-	-	-	-	-
Prior to PICU (days)	551†	-	-	1	0–4	44	-	-	3	0–24	508	-	-	0	0–2	0.004
After PICU (days)	552	-	-	6	3–12	44	-	-	7	3–14	508	-	-	5.5	3–12	0.099
Total hospitalisation (days)	552	-	-	12	7–25	44	-	-	20	10–41	508	-	-	12	7–23	0.003
**Hospitalisation outcome**	552	-	-	-	-	44	-	-	-	-	508	-	-	-	-	-
Deaths	552	69	12.5	-	-	44	8	18.2	-	-	508	61	12.0	-	-	0.236

CRE, carbapenem-resistant Enterobacterales; IQR, interquartile range; PICU, paediatric intensive care unit.

† , denominators differed because of missing data;

‡ , prevalent CRE.

### Children with prevalent Carbapenem-resistant Enterobacterales

Children who were CRE-colonised on PICU admission (44/552, 8%) were predominantly male (56.8%) with a median birthweight of 1910 g (IQR 1075 g – 2695 g). They were younger than CRE-non-colonised patients at PICU entry (median 4.5 months vs 10 months; *p* < 0.05), with approximately 80% (35/44) being younger than 1 year of age. The HIV exposure and infection proportions were similar to those of CRE-non-colonised children (22.7% and 4.5% vs 21.9% and 4.5% respectively, *p* > 0.05). Patients admitted to PICU after elective surgical admissions were at high risk of prevalent CRE if they had had a previous neonatal admission (13/52, 25%). Almost 75% of children with prevalent CRE (29/44) had a history of hospitalisation as neonates, a third (33%) of whom had required NICU admission. Thirty-six (81.8%) were hospitalised in the 12 months before PICU admission and 67% had received prior treatment with a carbapenem antibiotic. See Table 2 for all factors significantly associated with prevalent CRE.

**TABLE 2:** Factors significantly associated with prevalent Carbapenem-resistant Enterobacterales (all variables *p* < 0.05).

Variables	Total admissions *N* = 552					CRE positive on entry‡ *N* = 44† (8.0%)				CRE negative on entry *N* = 508† (92.0%)			
	*N* †	*n*	%	Median (days)	IQR (days)	*n*	%	Median (days)	IQR (days)	*n*	%	Median (days)	IQR (days)
Hospitalisation in prior 12 months	539	281	52.1	-	-	36	81.8	-	-	245	49.5	-	-
Neonatal admission	395	197	49.9	-	-	29	74.4	-	-	168	47.2	-	-
Premature	290	138	47.6	-	-	21	55.3	-	-	117	46.4	-	-
Neonatal surgical intervention	369	29	7.9	-	-	9	23.1	-	-	20	6.1	-	-
Neonatal intensive care admission	371	39	10.5	-	-	13	33.3	-	-	26	7.8	-	-
Duration of neonatal hospitalisation	187	-	-	16	7–34	-	-	30	16–54	-	-	14.5	7–30
Previous paediatric intensive care admission	550	72	13.1	-	-	15	34.1	-	-	57	11.3	-	-
Prior therapy with carbapenem	529	142	26.8	-	-	28	66.7	-	-	114	23.4	-	-

CRE, Carbapenem-resistant Enterobacterales.

† , Denominators differed because of missing data;

‡ , prevalent CRE.

### Children with incident Carbapenem-resistant Enterobacterales

Twenty-four children (10.1% of those with an exit swab, *N* = 237) became newly CRE-colonised during their PICU admission. They had a median age of 3 (IQR 1.5–7.5) months, 88% (21/24) were younger than 1 year of age and 52% were female. Median gestational age at birth was 36.5 (IQR 34.5–38) weeks and birthweight 2790 (IQR 2140–3100) gram.

Children in this group were most frequently admitted for pneumonia (12/24, 50%), sepsis/septic shock (5, 20.8%) and other respiratory conditions (4, 16.7%). Thirty per cent (7/24) had a positive blood culture during PICU admission: 3 (42.9%) for *K. pneumoniae* (2 extended-spectrum beta-lactamase-producing [ESBL], 1 CRE), and 2 (28.6%) for both *Stenotrophomonas maltophilia* and *Candida pelliculosa*.

Table 3 highlights the factors significantly associated with incident CRE. Prematurity, previous neonatal and NICU admission, neonatal surgical intervention and neonatal length of hospital stay were not associated with incident CRE. These results may be confounded by the limited number of exit CRE screens, especially in children who died. Thirteen per cent (3/24) of children with incident CRE died versus 3% (6/203) who were exit swab negative, *p* = 0.057.

**TABLE 3:** Factors significantly associated with incident Carbapenem-resistant Enterobacterales (all variables *p* < 0.05).

Variables	New CRE on exit† *N* = 24 (10.1%)				CRE negative on exit *N* = 203 (85.7%)			
	Median	IQR	*n*	%	Median	IQR	*n*	%
Age (months)	3	1.5–7.5	-	-	8	2–29	-	-
Duration of paediatric intensive care stay (days)	7	4–23	-	-	4	3–7	-	-
Total hospital stay duration (days)	25	15–56	-	-	14	9–30	-	-
Deaths at end of hospitalisation	-	-	4	16.7	-	-	9	4.4

CRE, Carbapenem-resistant Enterobacterales; IQR, interquartile range.

† , Incident CRE.

### Children with no exit Carbapenem-resistant Enterobacterales screen

Fifty-seven per cent (315/552) of children with an admission CRE screen did not have an exit swab. Contributors to this were non-repeat of positive screens (34 children, 10.6%, discussed under prevalent CRE), short duration of PICU admission (median 2 [IQR 1–3] days, exit screens were only performed after 48 h), as well as the high percentage of deaths in this group (49/315, 15.6%, exit swabs not routinely performed postmortem).

### Bacterial specimens and antibiotics

All 552 participants were prescribed antibiotics during their admission with 26.8% (142/529) having received a carbapenem antibiotic. Cephalosporins were the most frequently prescribed agents (52.4% of all patients). Children known with prevalent CRE were more likely to receive carbapenems (36.4% [16/44] vs 17.3% [88/508]) than those who were CRE-negative.

Three patients, two with prevalent CRE and one with a CRAB on tracheal aspirate, received colistin. One patient received combination therapy with tobramycin and colistin for a *P. aeruginosa* bacteraemia, and the other received colistin and tigecycline therapy for previous CRE isolated from multiple specimens. Another child received tigecycline for a *Serratia marcescens* bacteraemia. In all these cases, combination therapy was used with high-dose meropenem (40 mg/kg given as 8 hourly infusions) and a second agent. No children were treated with ceftazidime-avibactam as it was not available during the study period.

**FIGURE 2:**
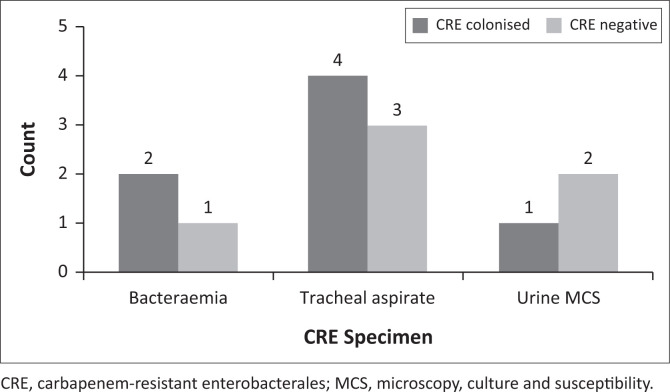
Carbapenem-resistant Enterobacterales-positive specimens by carbapenem-resistant enterobacterales status on paediatric intensive care admission.

**FIGURE 3:**
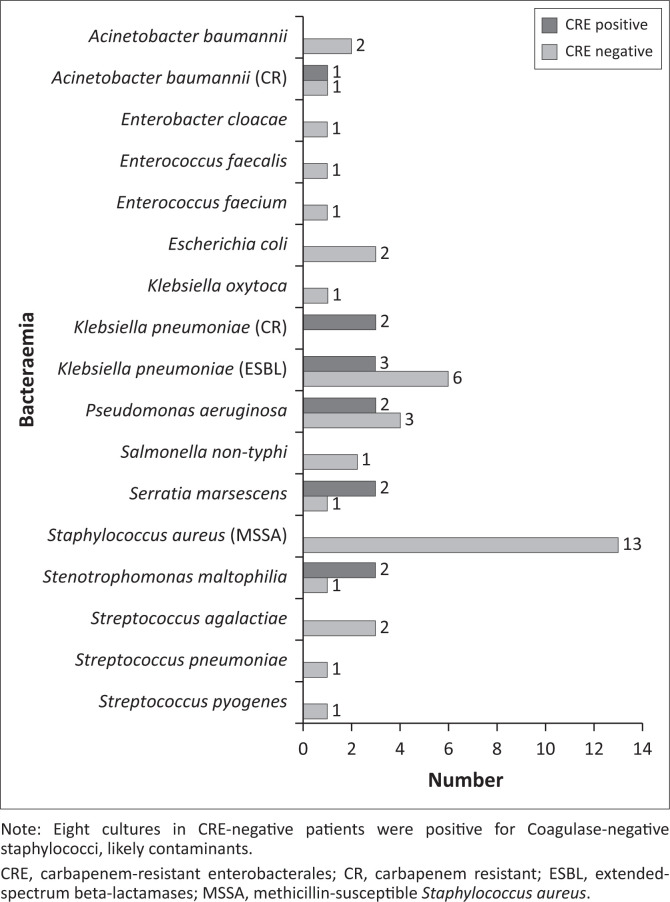
Bacteraemia by exit carbapenem-resistant enterobacterales status.

The most common bacteraemias in CRE-negative children were caused by methicillin-susceptible *Staphylococcus aureus* (MSSA) in 13/54 (24.1%) and ESBL *K. pneumoniae* in 8 (14.8%), while children with prevalent CRE experienced bacteraemia with *S. marcescens* (2/6, 33.3%) and *P. aeruginosa* (2, 33.3%). See Table 4 for more information on bacteraemia and antibiotics prescribed, Figure 2 for a review of carbapenem-resistant specimens and Figure 3 for a review of the positive blood cultures by exit CRE status.

**TABLE 4:** Bacteraemia and antibiotic profile by Carbapenem-resistant Enterobacterales colonisation status on paediatric intensive care admission.

Variables	Total screened admissions (*N* = 552)		CRE-positive on entry‡ (*N* = 44, 8.0%)		CRE-negative on entry (*N* = 508, 92.0%)		*p*
	*n*	%	*n*	%	*n*	%	
**Bacteraemia pathogens**	60	10.9	6	13.6	54	10.6	0.611
MSSA	13	21.6	-	-	13	24.1	-
*Serratia marcescens*	-	-	2	33.3	-	-	-
*Klebsiella pneumoniae* (ESBL)†	9	15.0	-	-	8	14.8	-
*Pseudomonas aeruginosa*	-	-	2	33.3	-	-	-
**Most frequent antibiotics received**							
3rd generation cephalosporin	289	52.4	17	38.6	271	53.3	-
Amoxicillin-clavulanic acid	108	19.6	-	-	100	19.7	-
Carbapenem	105	19.0	16	36.4	-	-	-
Piperacillin-tazobactam + Amikacin	-	-	9	20.4	-	-	-
Penicillins (Ampicilllin/Cloxacillin)	-	-	-	-	98	19.3	-

MSSA, methicillin-susceptible *Staphylococcus aureus*; CRE, carbapenem-resistant Enterobacterales; ESBL, extended-spectrum beta-lactamases.

† , Extended-spectrum beta-lactamase-producing;

‡ , prevalent CRE.

Seven per cent of children (40/552) were on treatment for tuberculosis: two had prevalent CRE and an additional three contracted incident CRE (5/40, 12.5% CRE positive on PICU discharge).

## Discussion

At TBH, a high proportion of children requiring PICU admission are CRE-colonised increasing the risk for CRE transmission in this resource-limited PICU with major space and staffing constraints. The prevalent CRE rate of 8% of PICU admissions was associated with known risk factors, including previous admission in the neonatal period. Incident CRE occurred in 10% of patients with an exit screening swab and was significantly associated with longer hospital stays, more frequent admissions, atypical and more complex to treat bacteraemia, as well as higher in-hospital mortality.

Paediatric critical care data on CRE prevalence is scarce. In an Egyptian PICU where 100 children received CRE screening swabs, the prevalence of Carbapenem-resistant Enterobacterales was 24%, with 80% exhibiting various carbapenemase genes.^17^ The prevalent CRE rate of 8% (44/552) in our cohort is high but more data are needed to accurately estimate this phenomenon at our institution and nationally. Factors associated with prevalent CRE in our cohort were previous neonatal admission, prematurity, neonatal surgical intervention, NICU admission, longer duration of neonatal hospitalisation, hospitalisation in the prior 12 months, previous PICU admission, as well as prior therapy with carbapenems. These are all in keeping with data from existing literature.^17^

In a prospective observational cross-sectional study in the PICU of Kalawati Saran Children Hospital, New Delhi, 10.7% of 300 patients who were previously CRE-negative children became CRE-colonised after 48 h in the PICU.^18^ This is similar to the 10.6% incident CRE rate we experienced in our unit. Factors significantly associated with incident CRE in our study were younger age at admission, extended duration of PICU and total hospital stay, as well as deaths at the end of hospitalisation (not in PICU).

Children with incident CRE had higher rates of bacteraemia than those who were CRE-negative (29% vs 23%, *p* = 0.015). Pathogens isolated from bacterial cultures differed markedly between the CRE-colonised patients (*K. pneumoniae* [3 ESBL and 2 CRE], *S. marcescens, P. aeruginosa* and *S. maltophilia*) and CRE-negative patients who mostly cultured MSSA.

Globally, *K. pneumoniae* has emerged as the most important multi-drug resistant (MDR) gram-negative organism.^19^ The prevalence and drug resistance of *Klebsiella* spp. have steadily increased both in hospital and in the community over the past decade, while the characteristics and distribution patterns of *E. coli* (previously the most common entity) have remained largely unchanged.^8^ This dominance of *K. pneumoniae* over other MDR gram-negatives as nosocomial pathogen is attributed to its adaptable genetic composition and large variety of ESBL enzyme types that facilitate antibiotic resistance and transmission of resistance to other bacterial species.^20,21^ This highlights the need for impeccable IPC measures when their presence has been detected. The Child Health and Mortality Prevention Surveillance (CHAMPS) network mortality data from seven African and Asian countries highlighted the impact of *K. pneumoniae* in under-five child deaths. These organisms contributed to 28.2% (155/549) of infectious deaths, 82% (127) were considered hospital-acquired.^22^ In our cohort, *K. pneumoniae* (9 ESBL and 2 CRE) also contributed 18% of bacteraemia: 12% in the CRE negative and 39% in CRE positive children (33% in prevalent CRE and 43% in incident CRE cases). Twenty-seven per cent of the children with *K. pneumoniae* bacteraemia died; all were CRE-negative on admission.

This study was strengthened by the fact that it included all 552 (86.5% of 638 total) children admitted to TBH PICU who received CRE screening swabs as standard of care. As all data are from a single unit, IPC measures remained consistent, but a significant limitation was our inability to fully isolate or cohort CRE-colonised or CRE-infected children because of staffing and spatial limitations. This may have contributed to higher rates of incident CRE. Another limitation and cause for potential bias is the lack of exit CRE screening in the most critically ill children with very short PICU stay because of demise before 48 h (26/58 (44.8%) of total deaths)) and poor exit CRE screening in children who died later in their admission. We speculate that CRE may have been present in some of these children and possibly contributed to their death. As all participants were admitted to PICU, which is a very specific subset of the paediatric population, our data are likely not generalisable to the community.

## Conclusion

Carbapenem-resistant Enterobacterales colonisation, both prevalent and incident, is common in PICU patients with implications for patient placement and empiric antibiotic treatment guidelines. A better understanding of the clinical predictors of CRE colonisation will support development of appropriate CRE screening recommendations and interventions.

In this era of increasing antibiotic resistance to even ‘last resort’ drugs, including colistin^10^ and ceftazidime-avibactam,^12^ the importance of CRE surveillance, infection prevention and control and antibiotic stewardship cannot be overstated. Novel protective and treatment strategies, like probiotic use in CRE-colonised patients,^10^ are important areas for future research.
